# Supplementary material for: Biodegradation and Metabolic Pathway of the Neonicotinoid Insecticide Thiamethoxam by Labrys portucalensis F11
Source: Int J Mol Sci. 2022 Nov 18;23(22):14326. doi: 10.3390/ijms232214326 (PMC9694413; doi:10.3390/ijms232214326)
Supplement: Supplementary file 1 [file ijms-23-14326-s001.zip › ijms-1975341-supplementary.pdf]

International Journal of *Molecular Sciences*

MDPI

Supplementary Information

Biodegradation and metabolic pathway of the neonicotinoid insecticide thiamethoxam by *Labrys portucalensis F11*

Oumeima Boufercha [1,2], Ana R. Monforte [3], Allaoueddine Boudemagh [2], António C. Ferreira [3], Paula M.L. Castro [3] and Irina S. Moreira [3*]

[1] Laboratory of Molecular and Cellular Biology, University of Brothers Mentouri, Constantine 1, Chaâbat Erssas Campus, Ain El Bey Road, Constantine 25000, Algeria. 1; boufercha.oumeima@yahoo.com
[2] Department of Microbiology, Faculty of Natural and Life Sciences, University of Brothers Mentouri, Con-stantine 1, Ain El Bey Road, Constantine 25000, Algeria. 2; boudemaghallaoueddine@yahoo.fr
[3] CBQF—Centro de Biotecnologia e Química Fina, Laboratório Associado, Escola Superior de Biotecnologia, Universidade Católica Portuguesa, Rua Diogo Botelho 1327, 4169-005 Porto, Portugal; amonforte@ucp.pt (A.R.M); asferreira@ucp.pt (A.C.F); plcastro@ucp.pt (P.M.L.C)

* Correspondence: ismoreira@ucp.pt

Table S1. Growth rate of *L. portucalensis* F11 in MSM media supplemented with TMX at the concentration 10.8 mg L^{-1}.

TMX supplementation	Growth rate (d^{-1})	R^2
TMX as sole carbon and nitrogen	0.0132 ± 0.0002	0.9911
TMX as sole carbon and sulfur	0.0097± 0.0006	0.9973
TMX as sole carbon	0.0198 ± 0.0002	0.9844
TMX + sodium acetate	0.0266 ± 0.0008	0.9943

Table S2. Growth rate of *L. portucalensis* F11 in in the presence of increasing concentration of TMX.

TMX Concentration (mg L^{-1})	TMX as sole carbon source		TMX with periodic feeding with acetate	
	Growth rate (d^{-1})	R^2	Growth rate (d^{-1})	R^2
37.3	0.0133 ± 0.0005	0.9902	0.0274 ± 0.0003	0.988
68.6	0.0056 ± 0.0002	0.997	0.0235 ± 0.0004	0.9886
128.7	0.0058 ± 0.0002	0.9955	0.0155 ± 0.0007	0.9856

Table S3. Growth rate of *L. portucalensis* F11 in MSM medium supplemented with periodic feeding in sodium acetate.

**MSM + sodium acetate**	**Growth rate (d^{-1})**	**R^2**
	0.0214± 0.0007	0.98

Table S4. Results of toxicity tests.

**Seed germination : *Lactuca sativa***		
**Experiment**	As sole carbon source	With periodic feeding with acetate
TMX= 10.8 mg L^{-1}	No effect	No effect
Degradation products	No effect	No effect
TMX= 37.4 mg L^{-1}	/	No effect
Degradation product	/	No effect
**Bioluminescence test : *Vibrio fischeri***		
TMX= 10.8 mg L^{-1}	19%	19%
Degradation product	0%	0%
TMX= 37.4 mg L^{-1}	/	28%
Degradation product	/	0%
**Toxi-chromo test: *E.coli***		
TMX= 10.8 mg L^{-1}	7.4%	7.4%
Degradation product	0%	0%
TMX= 37.4 mg L^{-1}	/	12.0%
Degradation product	/	0%

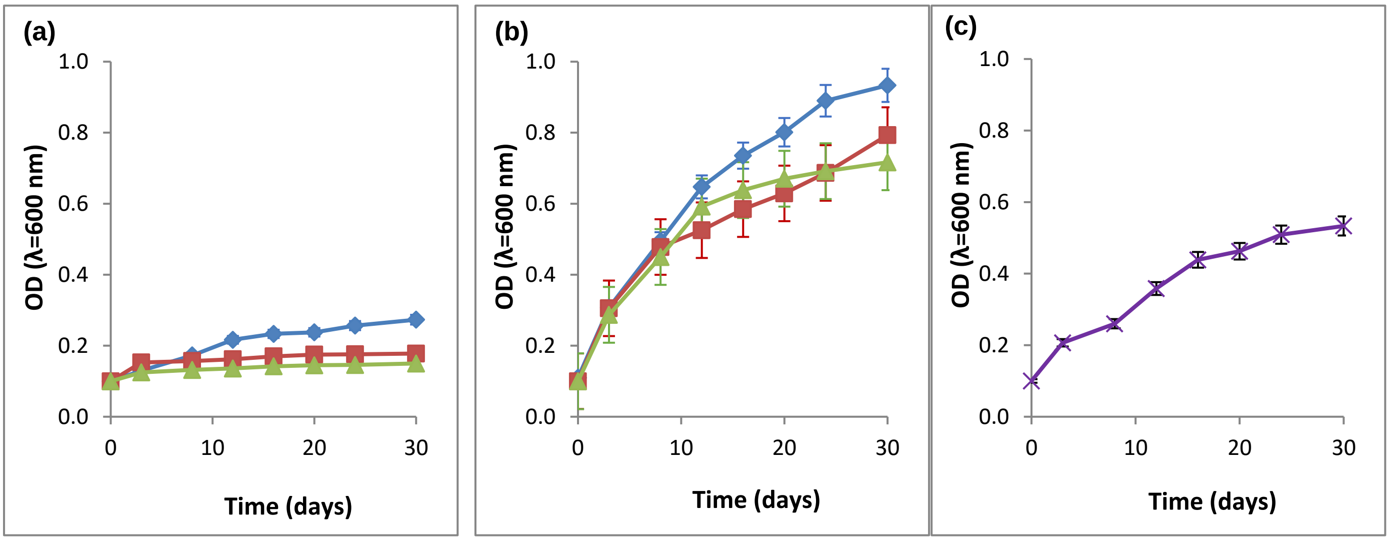

Figure. S1. Cell growth of *L. portucalensis* F11 during 30 days of incubation on MSM3 with TMX initial concentration: 37.4 mg L-1 (♦), 68.6 mg L-1 (■) and 128.7 mg L-1 (▲) **(a)** as sole carbon source and **(b)** with periodic feeding on sodium acetate. (c) Cell growth of *L. portucalensis* F11 with periodic feeding on sodium acetate without TMX. Error bars presented means of three replicates ± standard deviation.

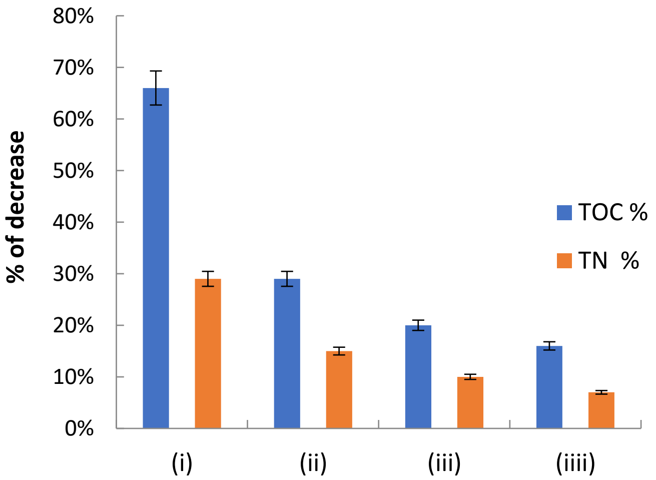

Figure.S2. Percentage of TOC and TN reduction during TMX degradation as sole carbon source: **(i)** 10.8 mg L-1, **(ii)** 37.4 mg L-1, **(iii)** 67.6 mg L-1 and, **(iiii)** 128.7 mg L-1.

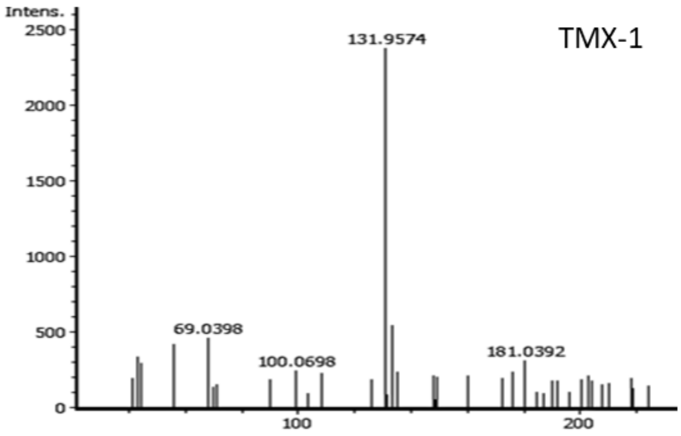
TMX-1
Intens.
131.9574
69.0398
100.0698
181.0392

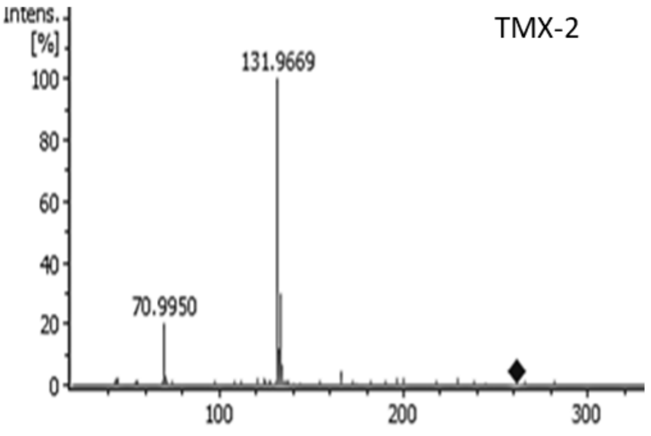
TMX-2
Intens.
[%]
131.9669
70.9950

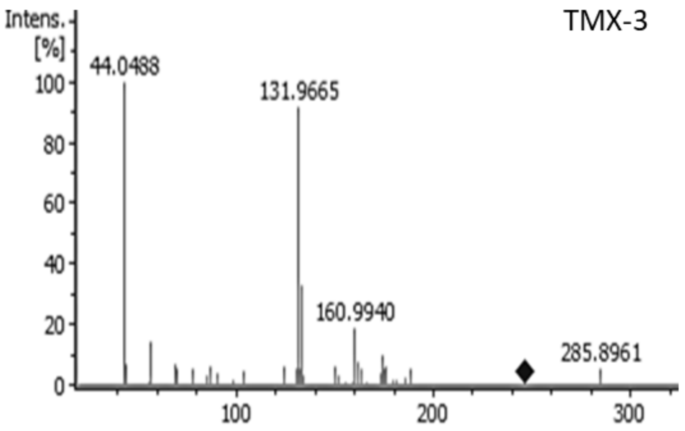
TMX-3
Intens.
[%]
44.0488
131.9665
160.9940
285.8961

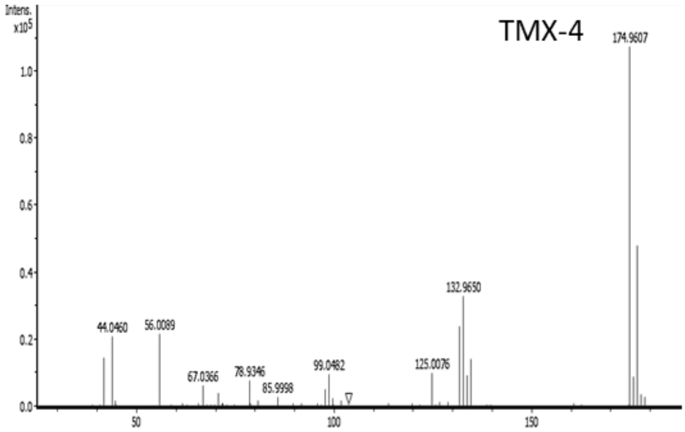
TMX-4
Intens.
x10^5
174.9607
132.9650
44.0460
56.0089
67.0366
78.9346
85.9998
99.0482
125.0076

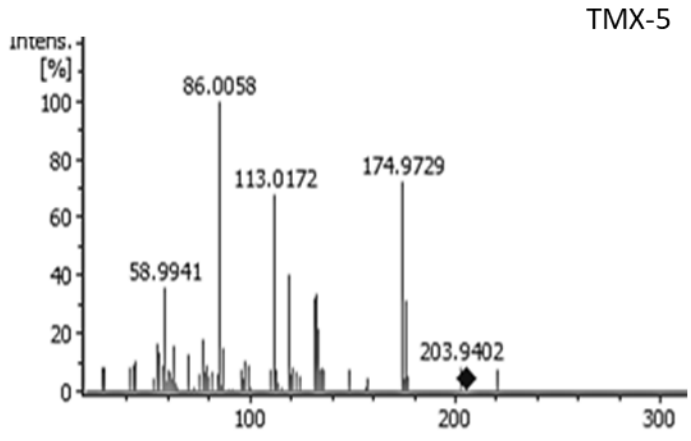
TMX-5
Intens.
[%]
86.0058
113.0172
174.9729
58.9941
203.9402

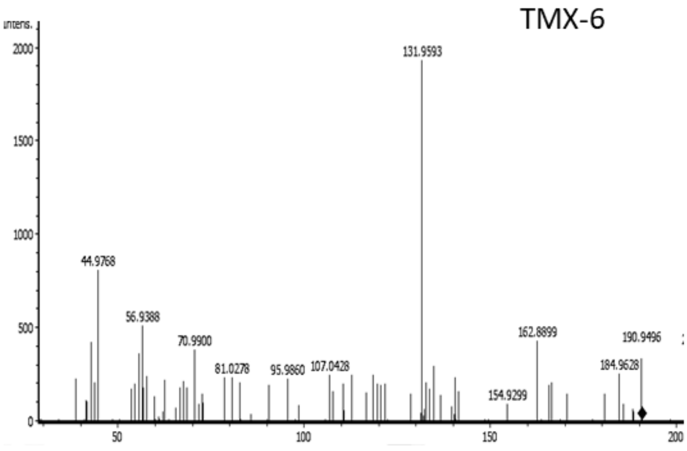
TMX-6
Intens.
131.9593
44.9768
56.9388
70.9900
81.0278
95.9860
107.0428
154.9299
162.8899
184.9628
190.9496

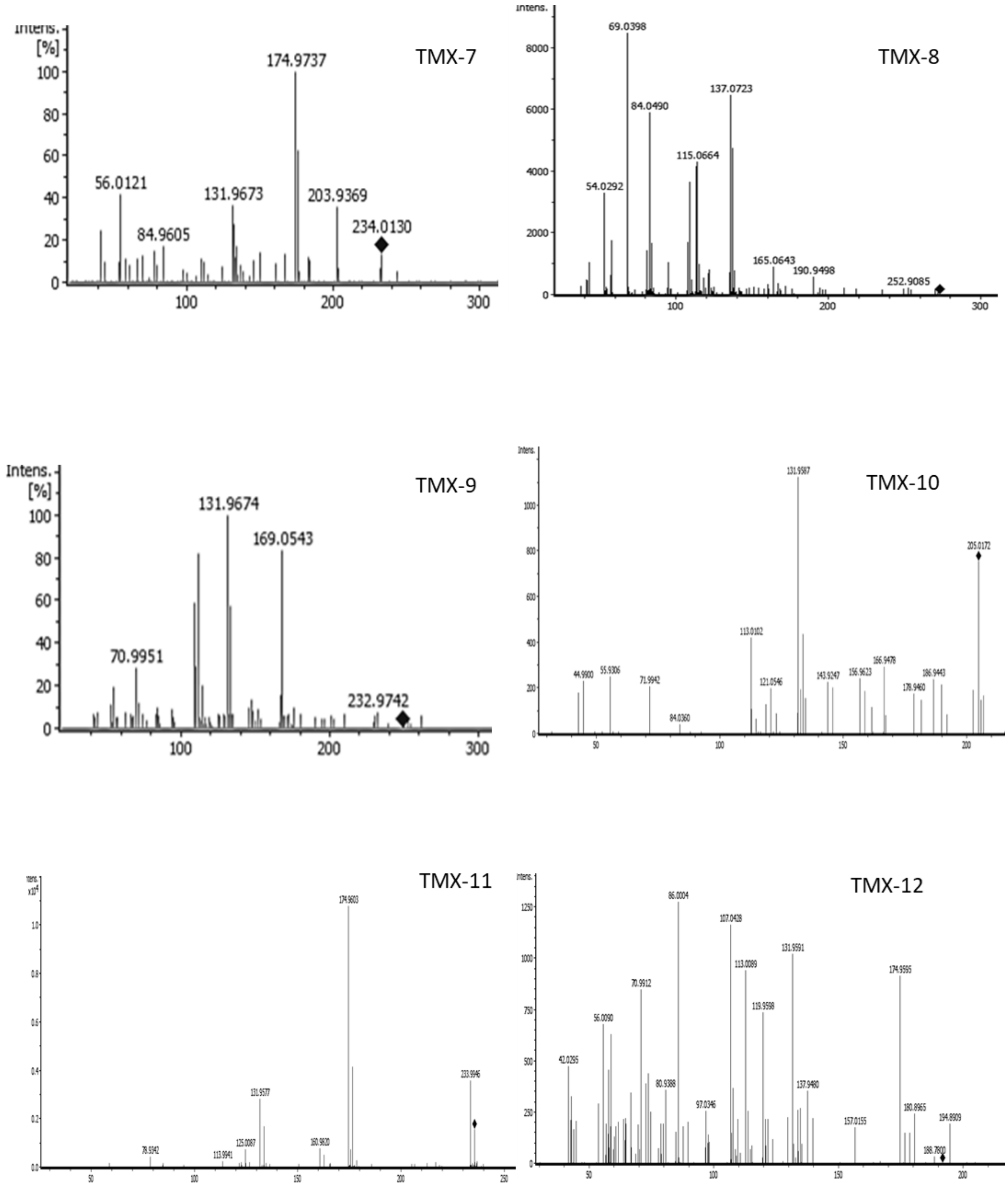

Figure S3. MS/MS spectra of the intermediate metabolites TMX1-TMX12.
